# Efficacy and safety of acupuncture combined with Chinese herbal medicine in the treatment of primary liver cancer: A protocol for systematic review and meta-analysis

**DOI:** 10.1097/MD.0000000000027497

**Published:** 2021-10-08

**Authors:** Shen Dong, Xiong Zhuang, Liu Yangyang, Leng Yan, Deng Houbo, Wang Song, Xiangtong Meng, Liu Tiejun

**Affiliations:** a Changchun University of Chinese Medicine, 1035 Bo Shuo Road, Changchun City, Jilin Province, China; b Department of Hepatology, First Affiliated Hospital to Changchun University of Chinese Medicine, 1478 Gongnong Road, Changchun City, Jilin Province, China; c Endocrinology, First Affiliated Hospital to Changchun University of Chinese Medicine, 1478 Gongnong Road, Changchun City, Jilin Province, China.

**Keywords:** acupuncture, Chinese herbal medicine, efficacy, meta-analysis, primary liver cancer, protocol, systematic review

## Abstract

**Background:**

Primary liver cancer (PLC) is a common cancer, and its morbidity and mortality are ranked 6th and 3rd in the world for malignant tumors, respectively. And this number is still on the rise, seriously endangering people's health. In recent years, acupuncture combined with Chinese herbal medicine have been widely used in the treatment of PLC, and there are few restrictions. However, we have not found a meta-analysis of their synergistic effects. Therefore, this systematic review and meta-analysis will evaluate the efficacy and acupuncture combined with Chinese herbal medicine in the treatment of primary liver cancer.

**Method:**

We will search the following databases from inception up to August 20, 2021: PubMed, Web of Science, Embase, AMED, Cochrane Library, CNKI, VIP, CBM, and Wanfang. There will be no restrictions regarding publication date or language. We will apply a combination of medical keywords and words, including “acupuncture,” “Chinese herbal medicine” and “primary liver cancer”. Additionally, we will manually search all reference lists from relevant systematic reviews to find other eligible studies. We will use the random effects model in REVMAN v5.3 for meta-analysis. The study for acupuncture combined with Chinese herbal medicine in the treatment of PLC was a randomized controlled study. Two researchers will independently review the research selection, data extraction, and research quality assessments. Finally, we will observe the outcome measures.

**Results:**

This study will provide evidence-based medical evidence for the treatment of PLC with a combination of acupuncture and Chinese herbal medicine, and provide new ideas and methods for the treatment of PLC.

Registration number: INPLASY202180103

## 1 Introduction

Primary liver cancer (PLC) is a common cancer, and its morbidity and mortality are ranked 6th and 3rd in the world for malignant tumors, respectively.^[1]^ According to statistics, there were 841,080 new cases and 781,631 deaths in 2018, accounting for 4.8% and 8.2% of all new cancer cases in the world, and this number is still on the rise, seriously endangering people's health.^[2]^ Although there are many treatment methods, such as surgical treatment, chemotherapy, interventional therapy, and molecular targeted therapy, they have many limitations in the scope of application (such as liver function Child-Push score and tumor staging). Moreover, the efficacy of the above treatments did not meet expectations, so the mortality and prognosis of PLC still have not been improved.^[3,4]^ In short, the difficult-to-treat characteristics of PLC have caused a major global health and economic burden.

In recent years, acupuncture combined with Chinese herbal medicine have been widely used in the treatment of PLC, and there are few restrictions.^[5]^ As an important part of traditional Chinese medicine, acupuncture has been confirmed to be effective in relieving various types of pain, and it has been used in the treatment and care of PLC and its complications.^[6,7]^ As a complementary and alternative medicine, Chinese herbal medicine has gradually shown advantages in the treatment of PLC, which is one of the current research hotspots.^[8,9]^ However, there is no obvious evidence to show the effectiveness of acupuncture and Chinese herbal medicine for PLC and the incidence of its side effects. This is also an important reason that prevents it from spreading to the Western world for its treatment of PLC. Therefore, this systematic review and meta-analysis will evaluate the efficacy and acupuncture combined with Chinese herbal medicine in the treatment of primary liver cancer.

## 2 Materials and methods

### 2.1 Information sources and search strategy

This research is based on preferred reporting items for systematic review and meta-analysis protocols (PRISMA-P).^[10]^ This study is a retrospective study and meta-analysis, so the study design, process, and results do not require patient and public participation or ethical approval. We will search the following databases from inception up to August 20, 2021: PubMed, Web of Science, Embase, AMED, Cochrane Library, CNKI, VIP, CBM, and Wanfang. There will be no restrictions regarding publication date or language. We will apply a combination of medical keywords and words, including “acupuncture,” “Chinese herbal medicine” and “primary liver cancer”. Additionally, we will manually search all reference lists from relevant systematic reviews to find other eligible studies. The expected registration has been approved by the International Platform of Registered Systematic Review and Meta-analysis Protocols. (https://inplasy.com/inplasy-2021-8-0103/). And the registration number is INPLASY202180103. The search strategy for the PubMed is presented in Table 1.

**Table 1:** The search strategy for the PubMed.

Number	Terms
#1	Primary liver cancer (all field)
#2	Hepatocellular carcinoma (all field)
#3	Intrahepatic cholangiocarcinoma (all field)
#4	Hepatocellular carcinoma - mixed intrahepatic cholangiocarcinoma (all field)
#5	Liver cancer (all field)
#6	#1 or #2–5
#7	Acupuncture (all field)
#8	Needling (all field)
#9	Acupoint (all field)
#10	Acupuncture treatment (all field)
#11	Scalp acupuncture (all field)
#12	Electro acupuncture (all field)
#13	Ear acupuncture (all field)
#14	Intradermal needling (all field)
#15	Auricular acupuncture (all field)
#16	Fire needling (all field)
#17	Catgut embedding (all field)
#18	#7 or #8–17
#19	Chinese medicine (all field)
#20	Traditional Chinese medicine (all field)
#21	Chinese herb medicine (all field)
#22	Proprietary Chinese medicine (all field)
#23	Chinese Herbs (all field)
#24	Chinese herbal (all field)
#25	#19 or #20–24
#26	randomized controlled trial (all field)
#27	randomly (all field)
#28	controlled clinical trial (all field)
#29	randomized (all field)
#30	random allocation (all field)
#31	supportive treatmen (all field)
#32	single-blind method (all field)
#33	double-blind method (all field)
#34	trials (all field)
#35	comparators
#36	allocation
#37	#26 OR #27–36
#38	#6 And #18 And #25 And #37

### 2.2 Inclusion and exclusion criteria

The inclusion criteria were as follows:

1. the study was a randomized controlled study;
2. the included patients had primary liver cancer;
3. the experimental group was acupuncture combined with Chinese herbal medicine, and the control group was the best supportive treatment.

The exclusion criteria were as follows:

1. metastatic liver cancer;
2. other treatments in the experimental group;
3. the control group was not the best supportive treatment, but chemotherapy, interventional treatment, etc;
4. the literature is not the type of included research.

### 2.3 Study selection

**Figure 1:**
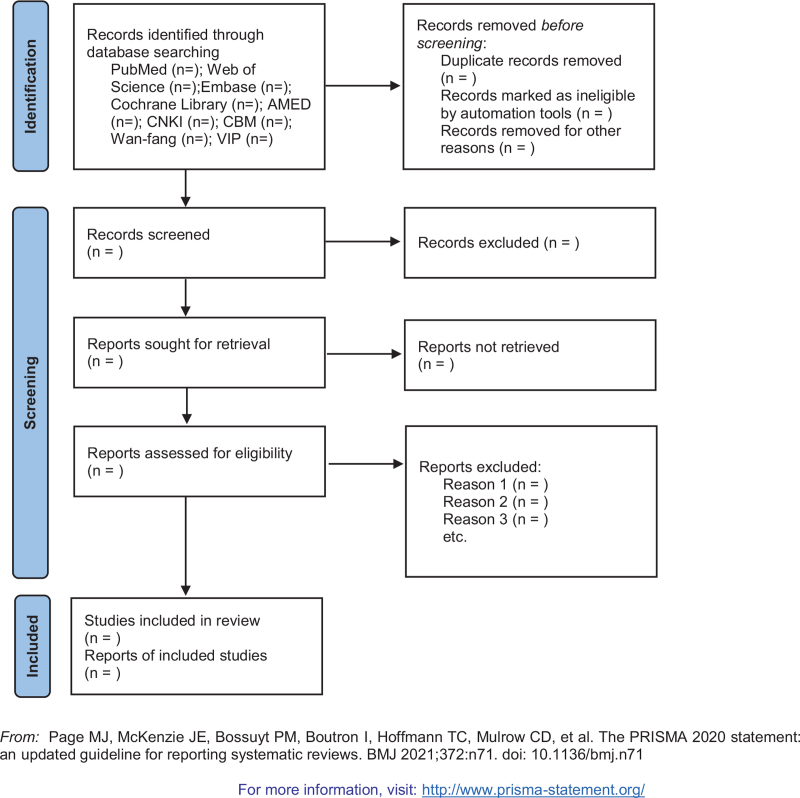
Flow diagram of study selection process.

Two researchers will independently review the eligibility of the data, and a third researcher will resolve any discrepancies. Then, the full texts will be screened in detail based on the above inclusion criteria. We will exclude all conference records, reviews, meta-analyzes, newspapers, guides, letters and other documents. During the research period, any disagreements between the authors will be resolved through discussion or negotiation with another researcher until a consensus is reached. The research selection process will be represented by the PRISMA flowchart.^[11]^ When the full text or the required information in the analysis process was missing, the author of the studies was contacted for data. The 2 authors will independently extract data according to the Cochrane manual guidelines and report the results in the PRISMA guidelines.^[12]^ Any differences will be resolved by consensus of all authors. A flowchart of the screening process is presented in Figure 1.

### 2.4 Assessment of study quality

The 2 authors will use the Cochrane risk bias assessment tool to separately assess the quality of randomized studies.^[13]^ The Cochrane bias risk assessment tool consists of 6 parts: selection bias (random sequence generation), selection bias (distribution hiding), implementation bias, measurement bias, follow-up bias, reporting bias, and other biases. Each item is divided into high-risk and low-risk. The 3 options are not clear. We will use Begg and Egger tests (set *P* < .1 to be statistically significant) and a funnel chart to assess publication bias. When the evaluation quality of the same study was inconsistent, it was resolved through consensus among all authors.

### 2.5 Outcome measures

#### 2.5.1 Main outcomes

The short-term curative effect is effective, the quality of life is stable, and the survival rate of patients is 6 months and 1 year.

#### 2.5.2 Additional outcomes

The occurrence of side effects, such as fever, nausea and vomiting, incidence of liver damage, etc.

### 2.6 Statistical analysis

We will use the random effects model in Review Manager software (REVMAN v5.3 Cochrane Collaboration) for meta-analysis, and *P* < .05 was considered statistically significant. Two authors will perform data extraction and input independently, the third author will check the data, and the other 2authors will perform data calculations. Evaluate the hazard ratio of the 95% confidence interval or the standardized mean difference of 95% CI for binary classification results or continuous results, respectively. We will use *I*^2^ statistics to detect clinical heterogeneity: 0% ≤ *I*^2^ < 25%, no heterogeneity; 25% ≤ *I*^2^ < 50%, mild heterogeneity; 50% ≤ *I*^2^ < 75%, moderate heterogeneity; *I*^2^ ≥ 75%, severe heterogeneity. If there was a high degree of heterogeneity between trials (*I*^2^ ≥ 50%), we tried to determine the source of heterogeneity through subgroup analysis, meta-regression and sensitivity analysis. Sensitivity analysis will be performed by omitting studies 1 at a time. We will use subgroup analysis based on different interventions, controls, and outcomes.

## 3 Discussion

Primary liver cancer is one of the most common cancers in the world. It has the characteristics of insidious onset and unobvious symptoms and has a poor prognosis. Generally, it has reached the middle and late stages of the disease when it is diagnosed, which seriously threatens human life and health.^[1–4]^ In the treatment of PLC, Chinese herbal medicine has the effects of relieving symptoms, prolonging survival and delaying the recurrence of PLC. Additionally, it can also reduce some of the side effects of Western medicine treatment and has a wide range of application prospects.^[14]^ Acupuncture also shows its own characteristics in the treatment of PLC and its complications.^[6,7]^ This study will provide evidence-based medical evidence for the treatment of PLC with a combination of acupuncture and Chinese herbal medicine, and provide new ideas and methods for the treatment of PLC.

## Acknowledgments

All the authors of this manuscript are very grateful to the various departments of Changchun University of Chinese Medicine for their support.

## Author contributions

**Conceptualization:** Shen Dong, Tiejun Liu.

**Data curation:** Xiong Zhuang, Liu Yangyang, Leng Yan.

**Formal analysis:** Xiong Zhuang, Wang Song, Xiangtong Meng.

**Funding acquisition:** Xiong Zhuang.

**Investigation:** Shen Dong, Xiong Zhuang, Wang Song, Xiangtong Meng.

**Methodology:** Shen Dong, Xiong Zhuang, Liu Yangyang, Deng Houbo.

**Project administration:** Leng Yan, Tiejun Liu.

**Resources:** Xiong Zhuang.

**Software:** Shen Dong, Xiong Zhuang.

**Supervision:** Leng Yan, Deng Houbo, Tiejun Liu.

**Validation:** Shen Dong, Leng Yan.

**Visualization:** Deng Houbo, Wang Song, Xiangtong Meng.

**Writing – original draft:** Shen Dong, Liu Yangyang, Leng Yan, Deng Houbo, Wang Song, Xiangtong Meng, Tiejun Liu.

**Writing – review & editing:** Shen Dong, Liu Yangyang, Leng Yan, Deng Houbo, Wang Song, Xiangtong Meng, Tiejun Liu.
